# Supplementary figures and images for: Complete genome sequence and annotation of the laboratory reference strain Shigella flexneri serotype 5a M90T and genome-wide transcriptional start site determination
Source: BMC Genomics. 2020 Apr 6;21:285. doi: 10.1186/s12864-020-6565-5 (PMC7132871; doi:10.1186/s12864-020-6565-5)

a)

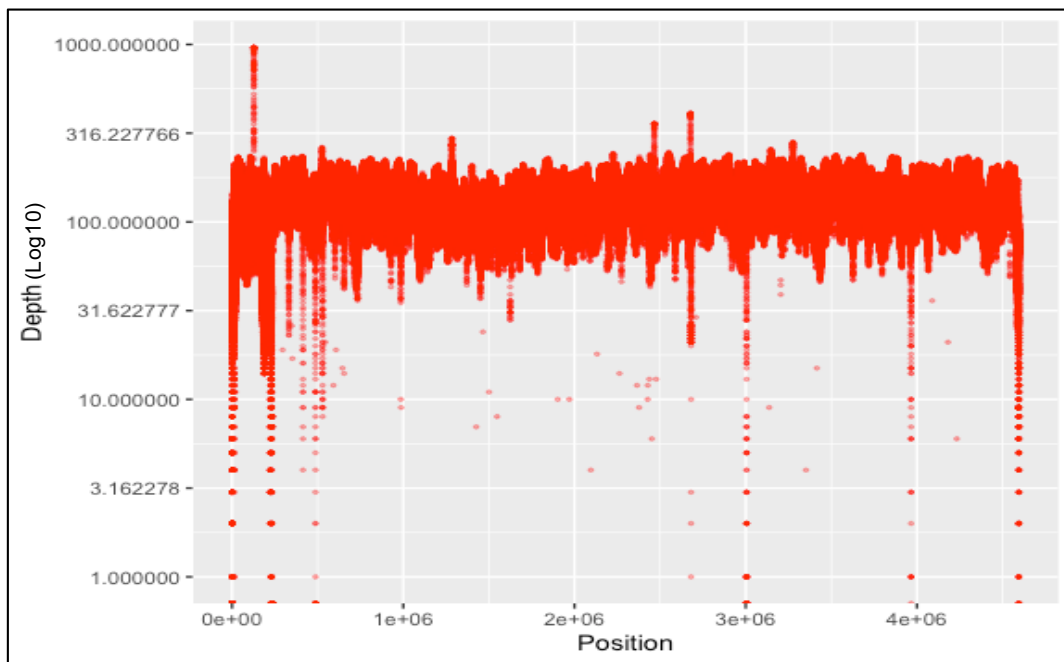

b)

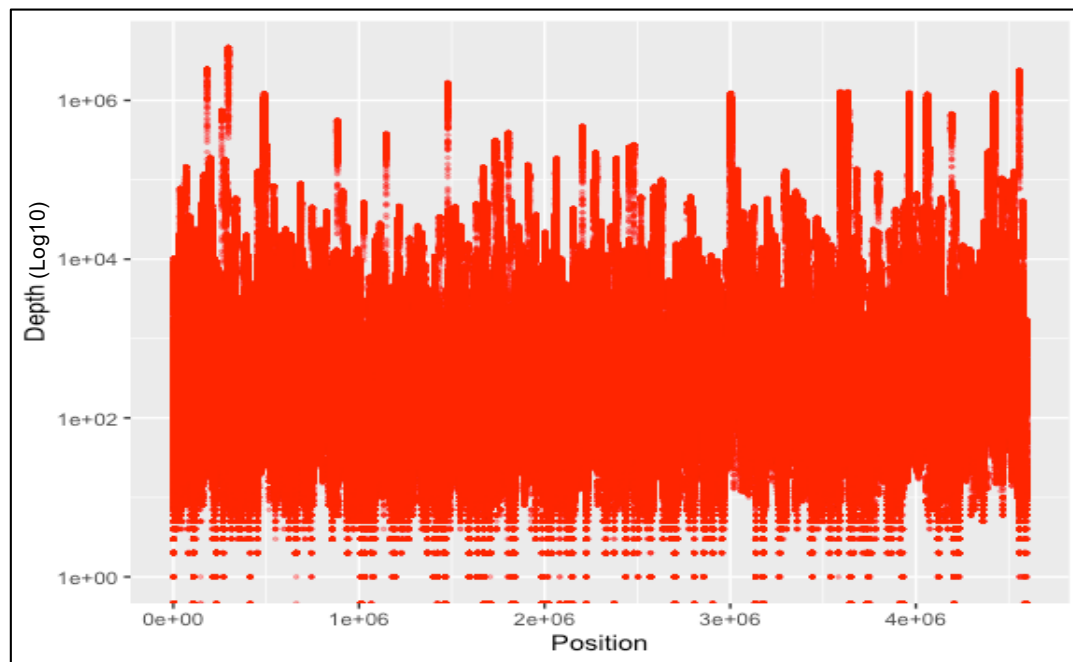

Supplement: Supplementary file 1 — Additional file 1: Figure S1. Genome coverage plot of S. flexneri 5a M90T with aligned DNA or RNA short-read sequences. a) Genome coverage of small DNA Illumina sequences. The percentage of genome covered with a depth of ≥5 is 99.98% with a mean coverage of 126X. b) Genome coverage of small RNA sequences. The percentage of genome covered with a depth of of ≥5 is 98.77% with a mean coverage of 989.9X. Coverage calculation was performed with Samtools depth [54, 55] using sorted alignments in BAM format as input. The figure was generated with ggplot2.3.2.1 in the R. 3.6.1 environment. [file 12864_2020_6565_MOESM1_ESM.pdf]
