# Supplementary material for: Complete genome sequence and annotation of the laboratory reference strain Shigella flexneri serotype 5a M90T and genome-wide transcriptional start site determination
Source: BMC Genomics. 2020 Apr 6;21:285. doi: 10.1186/s12864-020-6565-5 (PMC7132871; doi:10.1186/s12864-020-6565-5)
Supplement: Supplementary file 2 — Additional file 2: Table S1. Comparison of general features of the S. flexneri 5a M90T predicted with three different pipelines: Prokka [59], RAST [58] and PGAP/NCBI [60]. [file 12864_2020_6565_MOESM2_ESM.pdf]

	Prokka		RAST		PGAP/NCBI	
	Chromosome	pWR100	Chromosome	pWR100	Chromosome	pWR100
**Total length (bp)**	4596714	232195	4596714	232195	4596714	232195
**No. total CDSs**	4713	310	4943	364	4629	320
**No. Of rRNAs**	22	0	22	0	22	0
**No. of tRNAs**	103	0	102	0	102	0
**No. of pseudogenes**	0	0	0	0	640	129
